# Low‐Temperature Synthesis of SnO_2_ Nanocrystals as Electron Transport Layers for High‐Efficiency CsPbI_2_Br Perovskite Solar Cells

**DOI:** 10.1002/smsc.202200112

**Published:** 2023-01-29

**Authors:** Haoran Tian, Jingjing He, Xinyi Liu, Qing Li, Da Liu, Benben Shen, Shuang Yang, Qiang Niu, Yu Hou

**Affiliations:** ^1^ Key Laboratory for Ultrafine Materials of Ministry of Education Shanghai Engineering Research Center of Hierarchical Nanomaterials School of Materials Science and Engineering East China University of Science and Technology Shanghai 200237 China; ^2^ Inner Mongolia Erdos Electric Power and Metallurgy Group Company Limited Ordos Inner Mongolia 016064 China; ^3^ Shenzhen Research Institute of East China University of Science and Technology Shenzhen 518057 China

**Keywords:** electron transport, nanocrystals, perovskite solar cells, SnO_2_

## Abstract

Perovskite solar cells (PSCs) have recently become a hot topic in photovoltaics due to their high power conversion efficiency (PCE) and low‐cost processing. As a key component of PSCs, electron transport layers (ETLs) that play a vital role in efficient PSCs generally require high charge extraction ability, mobility, and easy fabrication. Herein, a simple route to obtain dispersion of tin oxide nanocrystals (SnO_2_ NCs) with uniform diameters and high stability as efficient ETLs for CsPbI_2_Br solar cells is demonstrated. The champion device achieves a remarkable PCE of 16.22% with an open‐circuit voltage of 1.30 V. This work offers a facile and effective way to fabricate high‐performance ETL nanocrystals in PSCs.

## Graphical abstract

A low‐temperature and controllable route to obtain dispersions of tin oxide nanocrystals (SnO_2_ NCs) with uniform diameters and high stability, as efficient electronic transport layers for CsPbI_2_Br solar cells, is reported. The champion device achieves a remarkable power conversion efficiency of 16.22% with an open‐circuit voltage of 1.30 V.

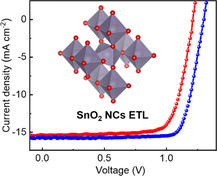

## 1 Introduction

Organic–inorganic lead halide perovskite solar cells (PSCs) have excellent properties and outstanding power conversion efficiencies (PCEs), owing to their physical characteristics.^[^ ^1^, ^2^, ^3^, ^4^, ^5^, ^6^ ^]^ In the past few years, the PCEs of PSCs have increased from 3.8%^[^ ^7^ ^]^ to 25.7%.^[^ ^8^ ^]^ However, the organic cation component, of methylamine (MA) and formamidine (FA), leads to affecting the device stability during heating, humidity, and sunlight illumination.^[^ ^9^, ^10^, ^11^, ^12^, ^13^, ^14^ ^]^ To overcome this issue, inorganic perovskite CsPbX_3_ (*X* = Cl, Br and I) solar cells have been widely studied in recent years.^[^ ^14^, ^15^, ^16^, ^17^, ^18^, ^19^, ^20^, ^21^ ^]^ Among inorganic perovskites,^[^ ^22^, ^23^, ^24^ ^]^ CsPbI_2_Br is regarded as a good candidate for its high efficiency and stability, owing to its suitable bandgaps (1.82–1.92 eV) and remarkable intrinsic phase stability.^[^ ^25^, ^26^, ^27^, ^28^ ^]^

Intensive researches were done in grain morphology and surface defect passivation of perovskite thin films.^[^ ^29^, ^30^, ^31^, ^32^, ^33^, ^34^ ^]^ On the other hand, energy loss at the interface or transport layers cannot be neglected.^[^ ^35^ ^]^ For a long period, TiO_2_ is the most widely used electron transport layer (ETL) material, but its low charge conductivity and high‐temperature process are adverse to acquiring efficient and stable PSCs. In comparison, SnO_2_ ETL has low‐temperature processability, high electron conductivity, high optical transmittance, and reasonable energy band for PSCs.^[^ ^36^, ^37^ ^]^ Numerous efforts have been made in the synthesis of SnO_2_ nanocrystals (SnO_2_ NCs) for PSC applications, including refluxing,^[^ ^38^, ^39^, ^40^ ^]^ hydrothermal,^[^ ^41^, ^42^ ^]^ solvothermal,^[^ ^43^ ^]^ microwave‐assisted,^[^ ^44^ ^]^ and room‐temperature solution stirring^[^ ^45^ ^]^ methods. Plentiful additives were used to regulate the synthesis of SnO_2_ nanocrystals, such as tetramethylammonium hydroxide (TMAH), tetrabutylammonium hydroxide (TBAOH), ammonium hydroxide (NH_3_·H_2_O), thiourea and so on. However, the controllable growth of SnO_2_ nanocrystals is still challenging to fully meet the requirements of PSCs.

Here, we developed a facile solution synthetic method of SnO_2_ colloid NCs using urea (CO(NH_2_)_2_) to control the formation kinetics of SnO_2_ nanocrystals. The as‐prepared SnO_2_ NCs can be fabricated as compact ETL films with superior conductivity, mobility, low trap density, and improved energy‐level alignment, which can dramatically promote the improvement of charge extraction and suppress nonradiative recombination of PSCs. The CsPbI_2_Br solar cells with the as‐synthesized SnO_2_ ETLs obtained an open‐circuit voltage (*V*_OC_) of 1.30 V and a PCE of 16.22%, respectively, which are much higher than that based on commercial SnO_2_ ETLs (*V*_OC_ = 1.21 V, PCE = 14.00%).

## 2 Results and Discussion

**Figure 1:**
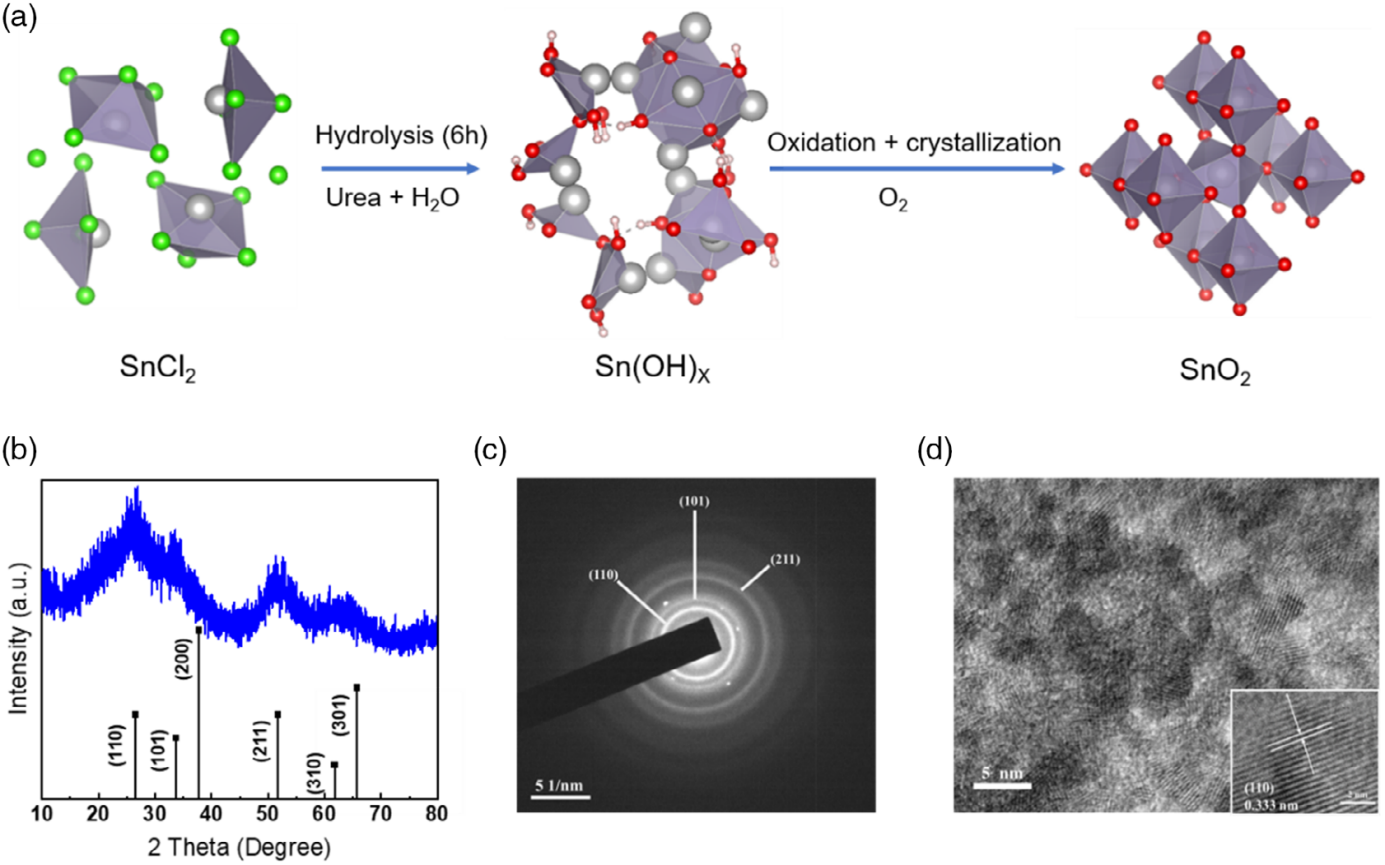
a) Schematic diagram of the proposed reaction mechanism. b) XRD pattern, c) SAED pattern, and d) TEM of u‐SnO_2_ NCs. The inset shows HRTEM images of one particle with the (110) lattice spacing of rutile SnO_2_.

We propose a synthetic mechanism of SnO_2_ NCs in **Figure** **1**a and S1, Supporting Information. First, the Sn(OH)^+^ intermediate species are produced after the exposure of SnCl_2_·2H_2_O in distilled water and the solution becomes milky. A sharp decrease in pH in the first 6 h reflects the hydrolysis of SnCl_2_·2H_2_O (Figure S2a, Supporting Information). Sn(OH)^+^ can be partially transformed back to Sn^2+^ due to the increase of H^+^ as the hydrolysis goes on. Notably, urea is decomposed into ammonium (NH_4_^+^) and cyanate (CNO^−^) ion, which is completely converted to NH_4_^+^ ions and carbon dioxide (CO_2_) with the consumption of hydrogen ion (H^+^),^[^ ^46^ ^]^ which explains the mild decrease of pH in the second stage of the reaction (Figure S2a, Supporting Information). The generated NH_4_^+^ ions with positive charge surround the as‐synthesized SnO_2_ NCs, which would contribute to the extreme stability of the colloid. After stirring for a few days, Sn(OH)^+^ is oxidized to Sn^4+^ by the dissolved oxygen in the solution.^[^ ^47^ ^]^ Figure S2b, Supporting Information shows the changes in dissolved oxygen levels as a function of reaction time. Due to the decomposition of urea, the existing hydroxide ion (OH^−^) reacts with Sn^4+^ to form Sn(OH)_4_, which is finally converted to SnO_2_ through dehydration reaction. However, due to the approximate ionic radii of Sn^2+^ (0.62 Å) and Sn^4+^ (0.69 Å), Sn^2+^ from the first step may be incorporated into the hexa‐coordinated SnO_2_ lattice.^[^ ^48^ ^]^ The presence of urea can control the reaction route and suppress the self‐doping of Sn^2+^ in SnO_2_ NCs.

**Figure 2:**
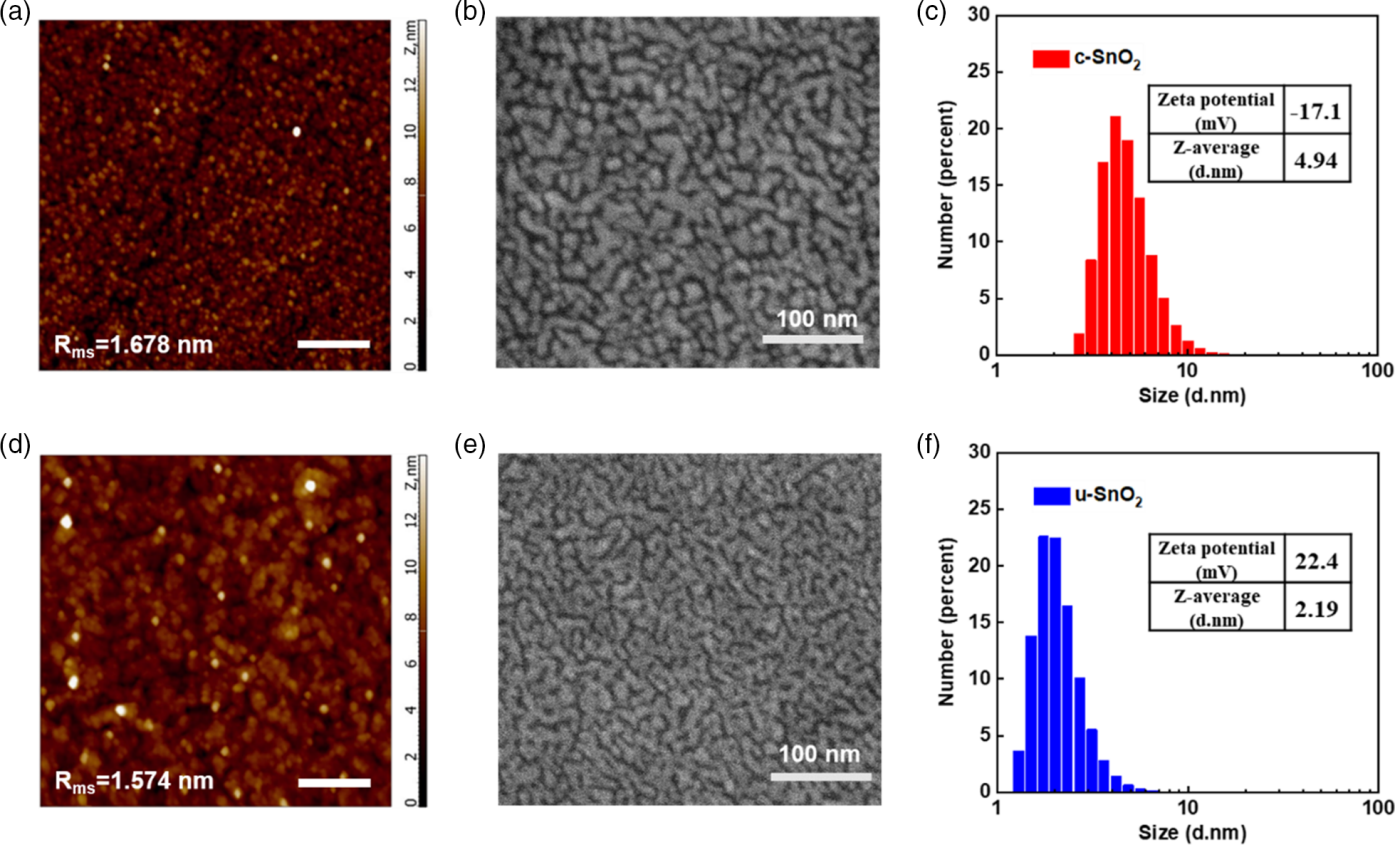
a,d) AFM images of c‐SnO_2_ (a) and u‐SnO_2_ (d) films with an area of 5 × 5 μm size; all scale bars are 1 μm. *R*_ms_ represents root‐mean‐square roughness of the film. b,e) SEM images of compact SnO_2_ films on ITO substrates: b) c‐SnO_2_ and e) u‐SnO_2_ NCs. c,f) Size distributions of c‐SnO_2_ (c) and u‐SnO_2_ (f) nanocrystal solutions measured by DLS analysis. The insets show the zeta potential and average particle size.

For convenience, we denote commercial SnO_2_ colloid dispersion and as‐synthesized SnO_2_ colloid dispersion as **c‐SnO**_**2**_ and u‐SnO_**2**_, respectively, in the following discussion. The X‐ray diffraction (XRD) pattern of u‐SnO_2_ NCs powder (Figure 1b) show diffraction peaks at 26.6, 33.8, 37.8, 51.8, 61.7, and 65.7°, which can be ascribed to tetragonal rutile SnO_2_. In Figure 1d, the transmission electron microscopy (TEM) image shows the extremely small nanocrystals, and most of them are between 3 and 5 nm in diameter. This result is in agreement with the dynamic light scattering (DLS) of u‐SnO_2_ NCs dispersion (2.19 nm), which is obviously smaller than c‐SnO_2_ (4.94 nm) (**Figure** **2**c,f). The zeta potentials of SnO_2_ NCs dispersions obtained by DLS analysis are −17.1 and 22.4 mV for the c‐SnO_2_ and u‐SnO_2_ NCs dispersion, respectively. The negative zeta potential for c‐SnO_2_ NCs corresponds to the presence of potassium hydroxide (KOH) stabilizer.^[^ ^49^ ^]^ In contrast, the positive zeta potential for u‐SnO_2_ should be caused by the hydrolysis of SnCl_2_ precursor. The larger absolute value of zeta potential for u‐SnO_2_ indicates more intense electrostatic repulsion between the charged SnO_2_ nanocrystals, which suggests better stability than c‐SnO_2_. High‐resolution TEM (HRTEM) confirms the high crystallinity of u‐SnO_2_ NCs and shows a nanocrystal with (110) lattice spacing (0.333 nm) of SnO_2_ with the tetragonal rutile structure. The selected‐area electron diffraction (SAED) pattern of u‐SnO_2_ NCs shows distinct electron diffraction circles indicating a polycrystalline nature (Figure 1c). X‐ray photoelectron spectroscopy (XPS) was implemented to analyze the surface chemical states of SnO_2_ film. The separation of 8.45 eV between the Sn3*d*5/2 and Sn3*d*3/2 levels shows that the SnO_2_ films only exists in a tetravalent oxidation state,^[^ ^50^ ^]^ and the shift of the Sn 3*d*5/2 state toward lower binding energy indicates that the u‐SnO_2_ has lower oxygen vacancy than c‐SnO_2_ (Figure S5, Supporting Information).

Compact SnO_2_ films were deposited onto indium tin oxide (ITO) substrates by the spin coating method. Atomic force microscopy (AFM) was used to study the surface morphology of the SnO_2_ films. Both films exhibit smooth surface with roughness of 1.574 and 1.678 nm for u‐SnO_2_ and c‐SnO_2_ films, respectively (Figure 2a,d). In addition, the scanning electron microscopy (SEM) images (Figure 2b,e) show the surface morphology of SnO_2_ films. Obviously, there are more cracks and holes on the surface of the c‐SnO_2_ film, while the surface of the u‐SnO_2_ film is smooth, uniform, and dense, which is beneficial to the subsequent deposition of perovskite films.

**Figure 3:**
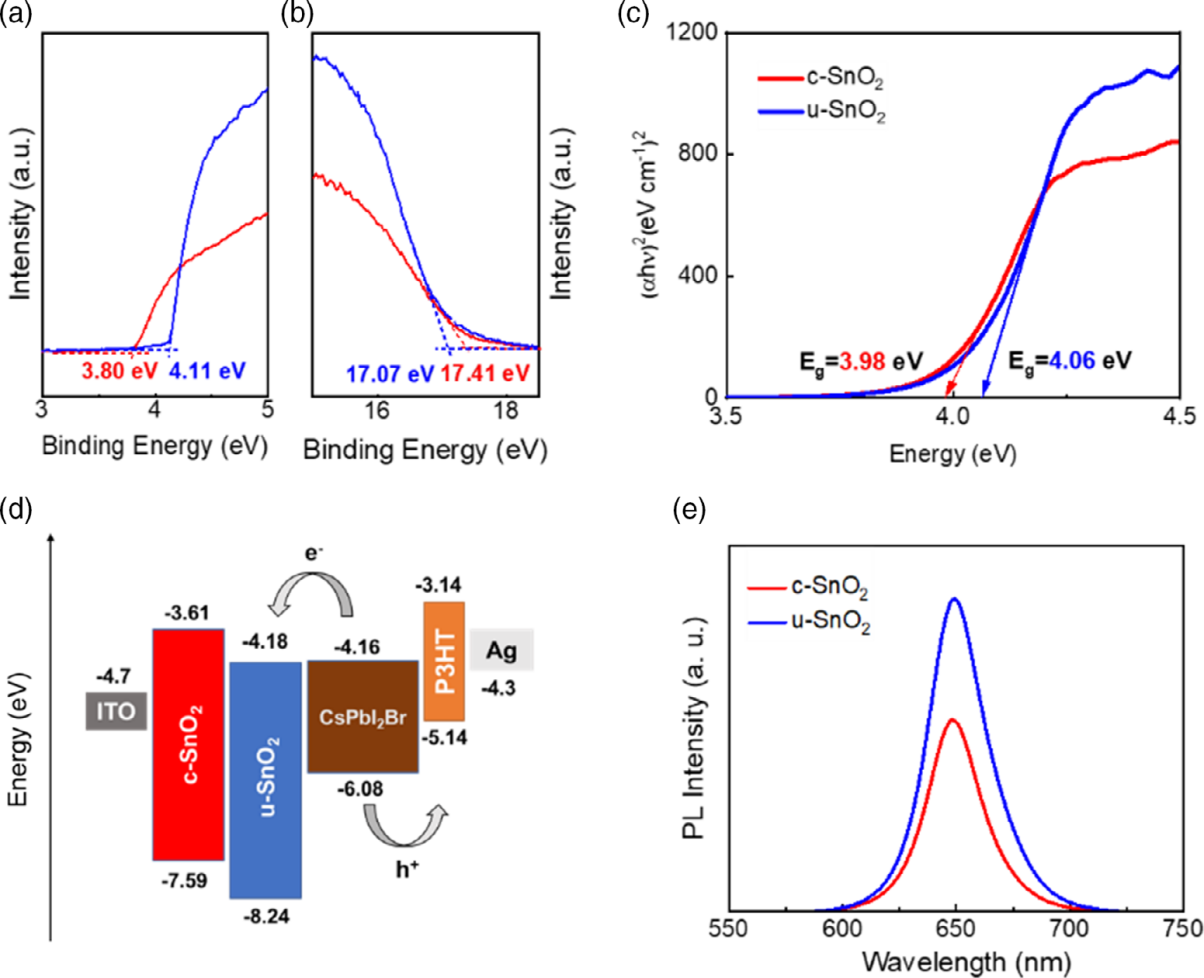
a,b) UPS and c) UV–vis spectra of c‐SnO_2_ and u‐SnO_2_ films. d) Energy‐level diagram of the CsPbI_2_Br PSCs based on c‐SnO_2_ film and u‐SnO_2_ film. e) Steady‐state photoluminescence (PL) spectra for CsPbI_2_Br perovskite films based on different ETLs.

We used ultraviolet photoelectron spectroscopy (UPS) to identify the energy band structure of the two ETLs (**Figure** **3**a,b). The deeper conduction band minimum (CBM) value of the u‐SnO_2_ film (−4.18 eV) ensures good electron extraction compared with that of the c‐SnO_2_ film (−3.61 eV). Meanwhile, the bandgaps of the c‐SnO_2_ film and the u‐SnO_2_ film were 3.98 and 4.06 eV according to the ultraviolet–visible (UV–vis) absorption spectra (Figure 3c), respectively. Owing to the larger bandgap of the u‐SnO_2_ film, its valence band maximum (VBM) (−8.24 eV) is much deeper compared to that of the c‐SnO_2_ film (−7.59 eV), which indicates the reduction of charge recombination in PSCs for u‐SnO_2_.^[^ ^51^ ^]^ The energy‐level diagram of CsPbI_2_Br PSCs with the c‐SnO_2_ film and the u‐SnO_2_ film is shown in Figure 3d. The better energy‐level alignment between the u‐SnO_2_ film and CsPbI_2_Br film would contribute to charge extraction. The CsPbI_2_Br film on u‐SnO_2_ ETL exhibits enhanced photoluminescence intensity (Figure 3e), indicating slow nonradiative recombination, which might be owing to fewer interfacial defects and lower surface roughness.

**Figure 4:**
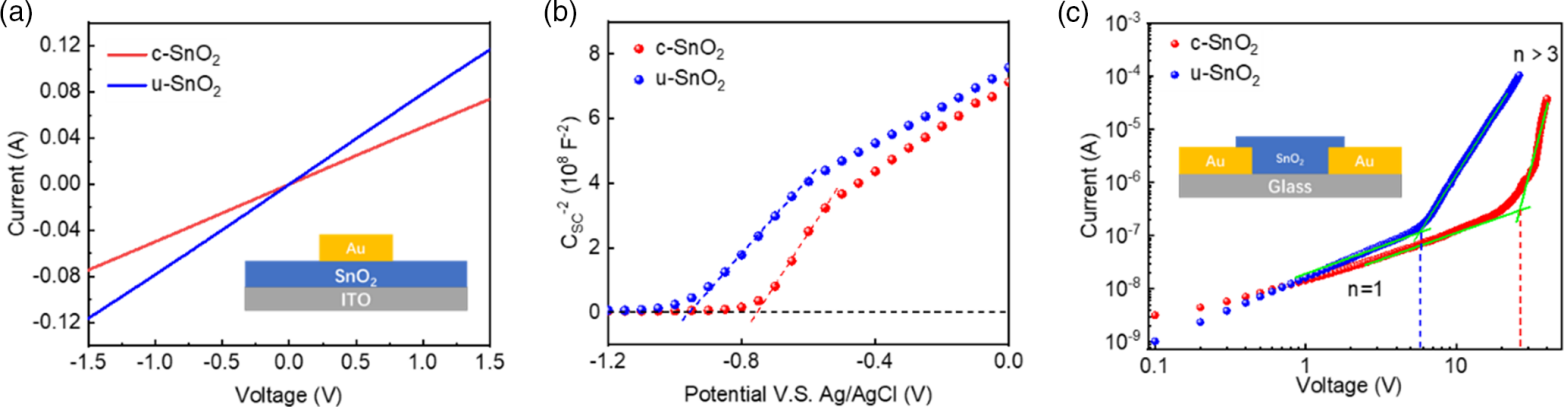
a) *I–V* curves of ITO/SnO_2_/Au structure. b) M–S plots of different SnO_2_ films. c) SCLC measurements of ITO/SnO_2_/Au devices.

To obtain excellent photovoltaic (PV) performance, high carrier density and mobility for ETLs are crucial in PSCs. Electrical conductivity tests of SnO_2_ ETLs are delivered based on structures of ITO/ETLs/Au. In **Figure** **4**a, the conductivity (*σ*) of u‐SnO_2_ and c‐SnO_2_ is calculated to be 1.48 × 10^−2^ and 0.95 × 10^−2^ mS cm^−1^. All detailed information is summarized in Table S1, Supporting Information. We fabricated a three‐electrode compartment with SnO_2_ films, an Ag/AgCl/3.5 m KHCO_3_ electrode and a platinum gauze as the working electrode, reference electrode, and counter electrode for *C*–*V* spectroscopy, respectively. Mott–Schottky (M–S) plots are shown in Figures 4b under a potential range of −1.5–0 V. The corresponding carrier density can be calculated using Equation (1) and (2)

(1) $$\frac{1}{C^{2}}=\frac{2}{q\varepsilon_{0}\varepsilon_{r}A^{2}N_{d}}\left(\varphi_{bi}-V-\frac{2kT}{q}\right)$$

(2) $$N_{d}=\frac{2}{q\varepsilon_{0}\varepsilon_{r}A^{2}\left[\frac{d\left(C^{-2}\right)}{dV}\right]}$$

where *φ*_b*i*_ is the built‐in potential, *q* is the elementary charge of an electron (1.6 × 10^−19^ *C*), *ε*_0_ is the permittivity of free space (8.85 × 10^−12^ F m^−1^), *ε*_r_ is the relative dielectric constant 9,^[^ ^52^ ^]^ *A* is the area of SnO_2_ film immersed in electrolyte (2.25 cm^2^), *C* is the interfacial capacitance, *V* is the applied voltage (*V*), and *N*_d_ is the carrier density. The carrier density of u‐SnO_2_ film (2.72 × 10^21^ cm^−3^) increased by ≈17% in comparison with c‐SnO_2_ film (2.33 × 10^21^ cm^−3^), which contributes to the improvement in conductivity of the relevant SnO_2_ ETLs. To estimate the trap density of ETLs, the space–charge‐limited current (SCLC) curves were obtained using lateral structured devices, which were constructed with two 100 μm‐gap Au interdigital electrodes (Figure 4c). The trap density (*N*) is calculated using Equation (3)

(3) $$N=\frac{4\varepsilon_{0}\varepsilon_{r}V_{\text{TFL}}}{\pi qL}$$

where *L* is the thickness of the ETL (100 μm), and *V*_TFL_ is the trap‐filled limit voltage.^[^ ^53^ ^]^ The trap density of the c‐SnO_2_ and u‐SnO_2_ films was calculated to be 1.68 × 10^10^ and 3.68 × 10^9^ cm^−2^, respectively. In general, the superior conductivity, large carrier density, and low trap density of the u‐SnO_2_ film suggest the faster charge transport and suppression of carrier accumulation at the ETL/perovskite interface, resulting in the excellent PV performance in the u‐SnO_2_‐based PSC. Dark current curves shown in Figure S6, Supporting Information indicate that the leakage current at the low‐voltage scale was weakened dramatically in the PSCs with u‐SnO_2_ ETL, which suggests the better interfacial contact between u‐SnO_2_ ETL and the active layer.

**Figure 5:**
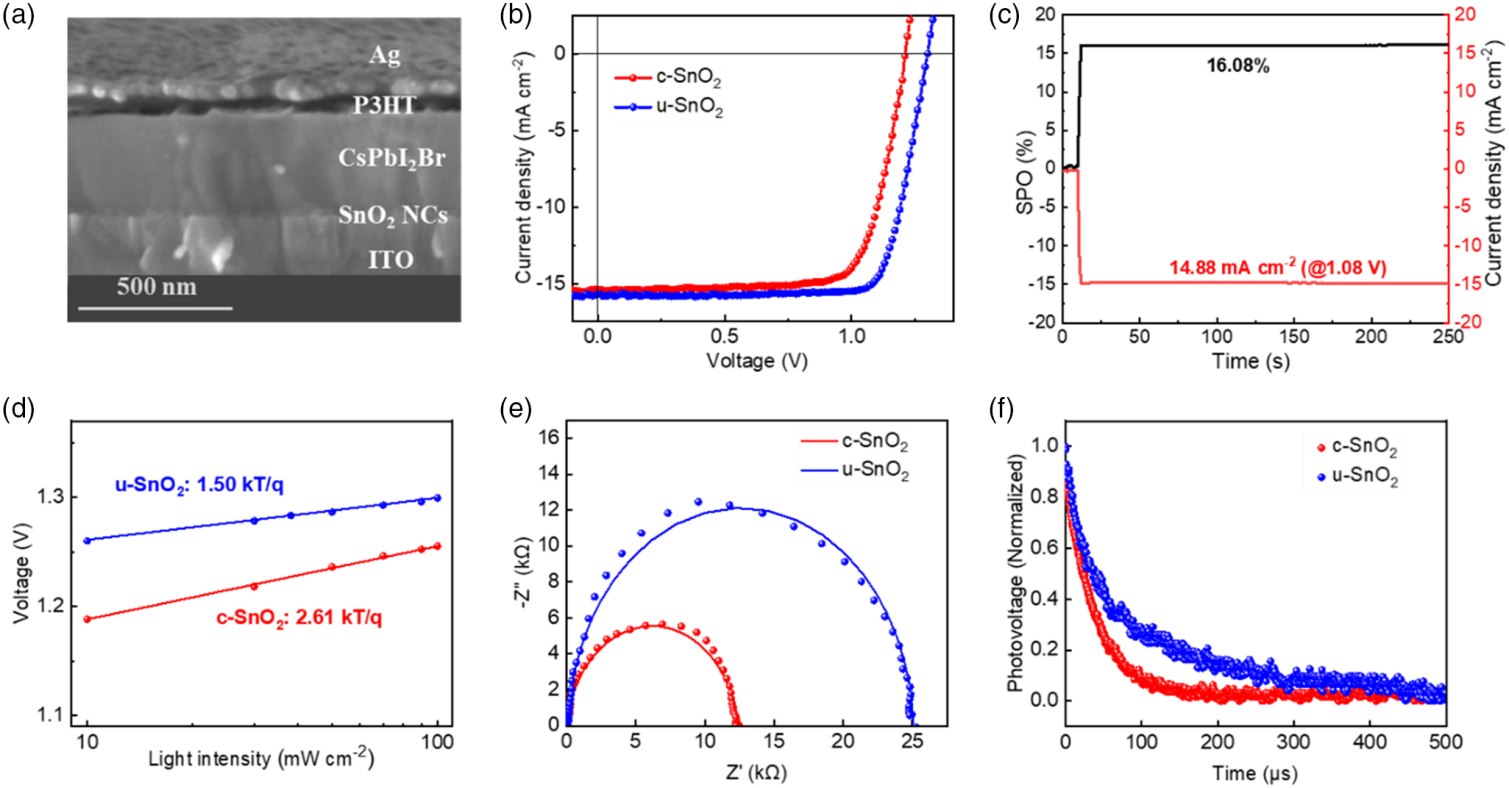
a) Cross‐sectional SEM image of PSC device with u‐SnO_2_ ETL. b) *J*–*V* curves of the champion PSC devices with c‐SnO_2_ and u‐SnO_2_ ETLs measured by reverse scan direction. c) The SPO and current density curves of the PSC device with u‐SnO_2_. d) *V*_OC_ as a function of light intensity. e) Nyquist plots and f) TPV decay curves of PSC devices with different ETLs.

PSC devices were fabricated with the structure of ITO/SnO_2_ NCs/CsPbI_2_Br/poly(3‐hexylthiophene2,5‐diyl) (P3HT)/Ag (**Figure** **5**a). The condition of perovskite films, which is relevant to crystallinity, grain size, and surface roughness, usually determines the performance of PSCs. XRD patterns of two perovskite films are shown in Figure S7, Supporting Information; two samples show the characteristic diffractions of α‐CsPbI_2_Br phase with two dominant peaks at 14.8° and 29.7°, corresponding to (100) and (200) planes, respectively. In Figure S8, Supporting Information, the SEM image of CsPbI_2_Br film coated on u‐SnO_2_ film shows larger grain size than that of c‐SnO_2_, and both of them display smooth surface. Figure 5b and Table S2, Supporting Information show the champion CsPbI_2_Br device based on u‐SnO_2,_ yielding a high *V*_OC_ of 1.30 V, a short‐circuit current density (*J*_SC_) of 15.68 mA cm^−2^, a fill factor (FF) of 0.80, and a remarkable PCE of 16.22%. In addition, the steady‐state power output (SPO) of the champion PSC under maximum power point (MPP) was 16.08%, with *J*_SC_ of 14.88 mA cm^−2^ for 250 s under a bias of 1.08 V (Figure 5c). In contrast, the c‐SnO_2_ device delivered a *V*_OC_ of 1.21 V, *J*_SC_ of 15.36 mA cm^−2^, FF of 0.75, and a PCE of 14.00%. The *J*_SC_ values from *J*–*V* curves agree well with the current density obtained from the external quantum efficiency (EQE) spectra (Figure S9, Supporting Information). The statistical comparison of *J*–*V* parameters of PSCs based on c‐SnO_2_ and u‐SnO_2_ is shown in Figure S10, Supporting Information. There is a significant increase in *V*_OC_ and FF from c‐SnO_2_ to u‐SnO_2_.

To evaluate the charge recombination of the PSCs, we conducted light intensity‐dependent *V*_OC_ and electrochemical impedance spectroscopy (EIS) measurement. Figure 5d shows the slopes of 2.61 and 1.50 kT/q for the PSCs with c‐SnO_2_ and u‐SnO_2_ ETLs, respectively. The smaller slope of the devices with u‐SnO_2_ indicates the lesser trap‐assisted recombination than that of the device with c‐SnO_2_.^[^ ^54^ ^]^ As shown in Figure 5e, the recombination resistance (*R*_rec_) of each PSC device is calculated from the diameter of the semicircle. Apparently, *R*_rec_ of PSC device with u‐SnO_2_ is almost doubled compared to that with c‐SnO_2_. In addition, it can be clearly seen that the PSC devices with u‐SnO_2_ exhibit higher *R*_rec_ value than those of the control ones, which indicates the remarkable decrease in undesirable recombination (Figure S11, Supporting Information). To further explore the relationship between carrier dynamics and device performance with different ETLs, we conducted the transient photovoltage (TPV) and transient photocurrent (TPC) measurements in PSC devices. TPV decay curves demonstrate that the PSC with u‐SnO_2_ ETL (76.1 μs) shows a longer lifetime than c‐SnO_2_‐based one (38.3 μs). The faster photocurrent decay response of u‐SnO_2_‐based device (0.9 μs) in Figure S12, Supporting Information reveals faster electron transport than c‐SnO_2_‐based one (2.0 μs).

Finally, we appraised the unencapsulated device stability with a device structure of ITO/SnO_2_/CsPbI_2_Br/P3HT/Au against heat. The thermal stability of CsPbI_2_Br devices was measured at 85 °C in N_2_‐filled glovebox in Figure S13, Supporting Information. In comparison with the ≈40% loss of PCE for the devices based on c‐SnO_2_ ETLs, the CsPbI_2_Br solar cells based on u‐SnO_2_ ETLs retained over 90% of its initial efficiency after 500 h, exhibiting excellent long‐term stability.

## 3 Conclusion

We have reported a synthetic method for the preparation of colloidal tin oxide nanocrystals with uniform particle size and good crystallinity. The as‐synthesized SnO_2_ nanocrystalline films have superior electrical conductivity, optimized band edges, and low trap density compared to commercial ones. The champion device based on u‐SnO_2_ film attained a high PCE of 16.22% with a *V*_OC_ of 1.30 V of CsPbI_2_Br cells. Our work will provide new insights into the design and synthesis of novel charge transport materials for ETLs in PSCs.

## Conflict of Interest

The authors declare no conflict of interest.

## Supporting information

Supplementary Material

## Data Availability

The data that support the findings of this study are available from the corresponding author upon reasonable request.
