# Supplementary material for: Low‐Temperature Synthesis of SnO2 Nanocrystals as Electron Transport Layers for High‐Efficiency CsPbI2Br Perovskite Solar Cells
Source: Small Sci. 2023 Jan 29;3(4):2200112. doi: 10.1002/smsc.202200112 (PMC11935836; doi:10.1002/smsc.202200112)
Supplement: Supplementary file 1 — Supplementary Material [file SMSC-3-2200112-s001.pdf]

Supporting Information

Low-temperature synthesis of SnO_2 Nanocrystals as Electron Transport Layer for High-Efficiency $CsPbI_2Br$ Perovskite Solar Cells

Haoran Tian, Jingjing He, Xinyi Liu, Qing Li, Da Liu, Benben Shen, Shuang Yang, Qiang Niu, and Yu Hou**

Experimental Section

1. Chemicals

Lead bromide ($PbBr_2$, 99.9%), lead iodide (PbI_2, 99.9%), cesium iodide (CsI, 99.9%), dimethyl sulfoxide (DMSO, 99.8%), chlorobenzene (99.9%), acetonitrile (99.8%), 4-tert-butylpyridine (t-BP, 96%), bis (trifluoromethane) sulfonimide lithium salt (Li-TFSI, 99.95%) and tin([illegible]) chloride dihydrate ($SnCl_2{\cdot}2H_2O$) were purchased from Sigma-Aldrich. Tin (IV) oxide (15% in H_2O colloidal dispersion) was purchased from Alfa Aesar. Poly (3-methylthiophene) (P3HT) was purchased from Xi'an Polymer Light Technology Corp. Urea ($CO(NH_2)_2$) was purchased from Sinopharm Chemidical Reagent Co. Indium-tin oxide (ITO) substrates (8 Ω per square) were purchased from Nippon Sheet Glass. All chemicals and solvents were used as received without further purification.

2. Preparation of Solutions and Devices

2.1. Preparation of Colloidal SnO_2 NPs Solution

676.95 mg of $SnCl_2{\cdot}2H_2O$ and 180 mg of $CO(NH_2)_2$ were dissolved in 20 mL deionized water in an open beaker under vigorous magnetic stirring at room temperature, forming a milky suspension. After continuous stirring for 4–5 d, a yellow and clear colloidal **u-SnO_2** NPs solution was obtained. As for **c-SnO_2** NPs solution, the SnO_2 colloidal dispersion obtained from Alfa Aesar (tin(IV) oxide, 15% in H_2O colloidal dispersion) was diluted to the concentration of 3 wt% using deionized water. Both obtained SnO_2 NPs solutions were filtered with 0.23 µm PTFE filter before using.

2.2. Device Fabrication

The patterned ITO substrates were washed by ultrasonication with soap, deionized water, acetone and isopropanol, respectively, for 30 min, then dried by nitrogen flow and finally treated with ultraviolet

ozone cleaner for 30 min. The obtained SnO_2 NPs solutions were spin-coated onto the glass/ITO substrates at 3000 rpm for 30 s in ambient air, followed by annealing at 150 °C in muffle furnace with the heating rate of 2 °C min^{-1} for 30 min. After cooling to room temperature, the substrates were treated with ultraviolet ozone for 15 min and then transferred to the nitrogen-filled glovebox. Subsequently, 40 µL $CsPbI_2Br$ precursor was loaded onto the substrate and spin-coated via a two-step process with 500 rpm for 3 s and 3500 rpm for 30 s. The $CsPbI_2Br$ layer was obtained by annealing the precursor film at 43 °C for 2 min and 160 °C for 10 min. P3HT transport layer was deposited onto the $CsPbI_2Br$ film by spin coating 20 µL P3HT solution at 2500 rpm for 25 s and followed by annealing at 120 °C for 10 min on a hotplate. Finally, the device was finished by evaporating the Ag or Au layers.

3. Characterization

The crystallographic information of SnO_2 nanocrystals powder was investigated by X-ray diffraction (XRD, Bruker Advance D8 X-ray diffractometer, Cu Kα radiation, 40 kV). The morphology and crystallization information of the SnO_2 nanoparticles were obtained by transmission electron microscope (TEM; JEOL, JEM-2100, 200 KV). Filed emission scanning electron microscopy (FESEM, HITACHI S4800) and atomic force microscopy (AFM, Veeco/DI) were used to characterize the morphology and roughness of the SnO_2 ETLs. X-ray photoelectron spectroscopy (XPS, PHI5300, Mg anode, 250 W, 14 kV) was used to analyze the elemental composition of the SnO_2 films. Work function of the SnO_2 films were measured by ultraviolet photoelectron spectrum (UPS) with He source of incident energy of 21.21 eV (He l line). The electrical conductivity tests are conducted based on the structure of ITO/ ETLs/Au under dark condition. Mott-Schottky plots of different SnO_2 films were performed using an electrochemical workstation (CHI 660E) in a three-electrode compartment with the 0.1 M $KHCO_3$ as the electrolyte, the SnO_2 films as the working electrode, an Ag/AgCl/3.5 M $KHCO_3$ electrode as the reference electrode and a platinum gauze as the counter electrode. Mott-Schottky data was recorded at the frequency of 1 KHz in the applied voltage range from -1.5 V to 0 V with an AC amplitude of 5 mV. Lateral structured devices were fabricated with two 100-µm-gap Au electrodes for the lateral electrical measurement. Transmittance spectra of different SnO_2 films were measured by using a Cary 500 UV-Vis-NIR Spectrophotometer. Photoluminescence (PL) measurement was acquired at room temperature using the spectrophotometer (Ocean optics QE pro) with an excitation wavelength of 365 nm. Cross-sectional SEM image of the PSC device was characterized by field emission scanning electron microscopy (FESEM, HITACHI S4800). Transient photovoltage (TPV) measurements were conducted by illuminating the devices under 1 sun intensity with a 337 nm laser pulses (SRS NL 100 Nitrogen Laser, frequency of 10 - 20 Hz, < 3.5 ns width) from the semitransparent electrodes, and the pulse laser generated photovoltage was recorded using a digital storage oscilloscope (Keysight, 1 GHz Agilent DSO7104B). Transient photocurrent (TPC) response was recorded

by digital storage oscilloscope (Keysight, 1 GHz Agilent DSO7104B) (the internal impedance of the oscilloscope was set to 50 Ω), and the device was illuminated with a 337 nm pulse laser (SRS NL 100 Nitrogen Laser, frequency of 10 – 20 Hz, < 3.5 ns). Solar cells were illuminated by a solar light simulator (Solar IV-150A, Zolix) and light intensity was calibrated using a standard Newport-calibrated KG5-filtered Si reference cell. The current density–voltage (*J*–*V*) curves of devices were measured with Keithley 2400 digital source meter under AM 1.5G irradiation (100 mW cm^{-2}) at a scan rate of 0.15 V s^{-1}(voltages scan range: -0.3 to 1.5 V, voltage step of 10 mV) in ambient environments. The devices were masked with a metal aperture to define the active area to be 0.0625 cm^2. The steady-state photocurrent output of the best performing devices was measured by biasing the device at the maximum power point (MPP) in ambient environments. Electrochemical impedance spectroscopy (EIS) was performed out using an electrochemical workstation (Parstat 2273, Princeton) in the frequency range of 1 MHz to 1 Hz under different positive bias voltages under dark conditions in air. EQE measurement was carried out on a Newport-74125 system (Newport Instruments) in air.

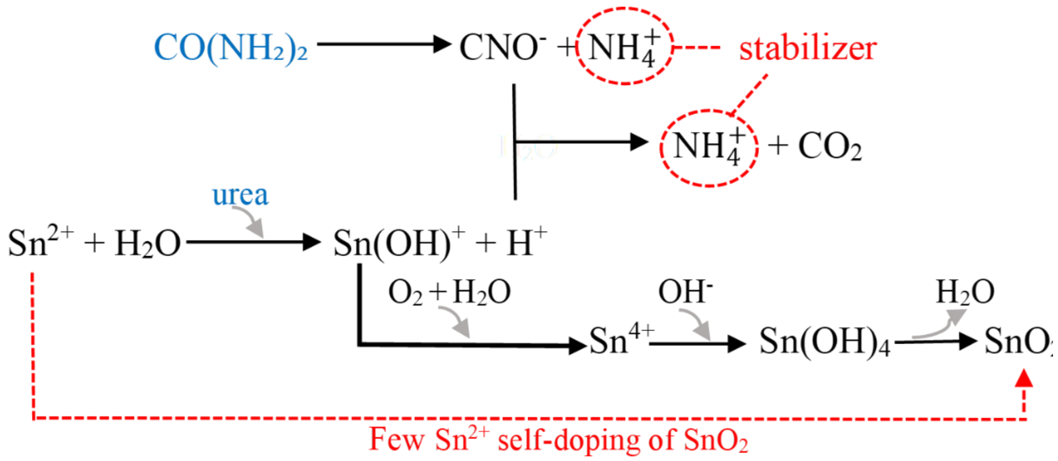

Figure S1. Schematic illustration of the formation mechanism of the u-SnO_2 NCs.

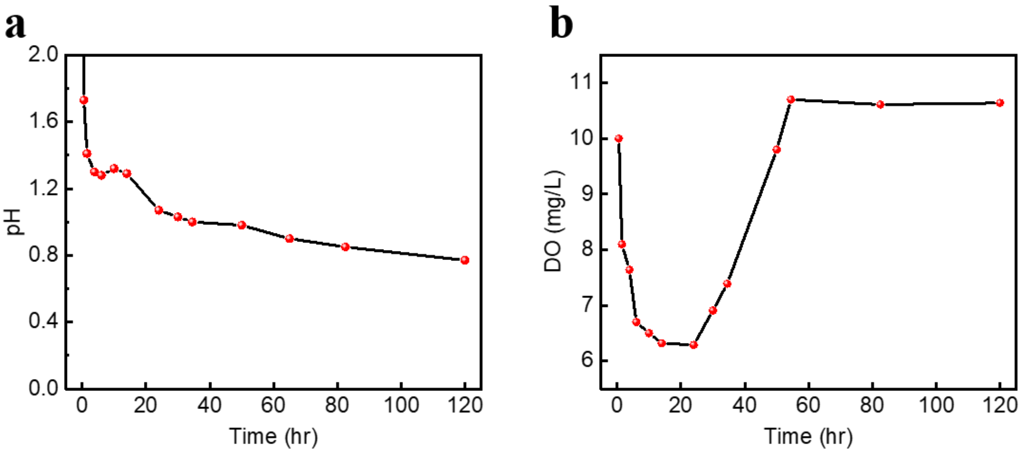

Figure S2. (a) Plot of pH versus reaction time. (b) Plot of dissolved oxygen (DO) versus reaction time.

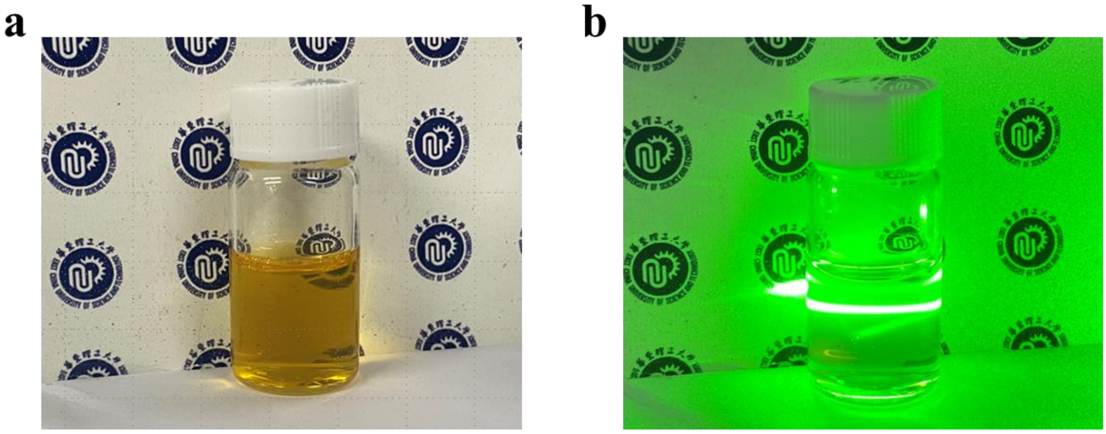

Figure S3. Digital images of u-SnO_2 NC solutions (a) under natural light and (b) green laser light.

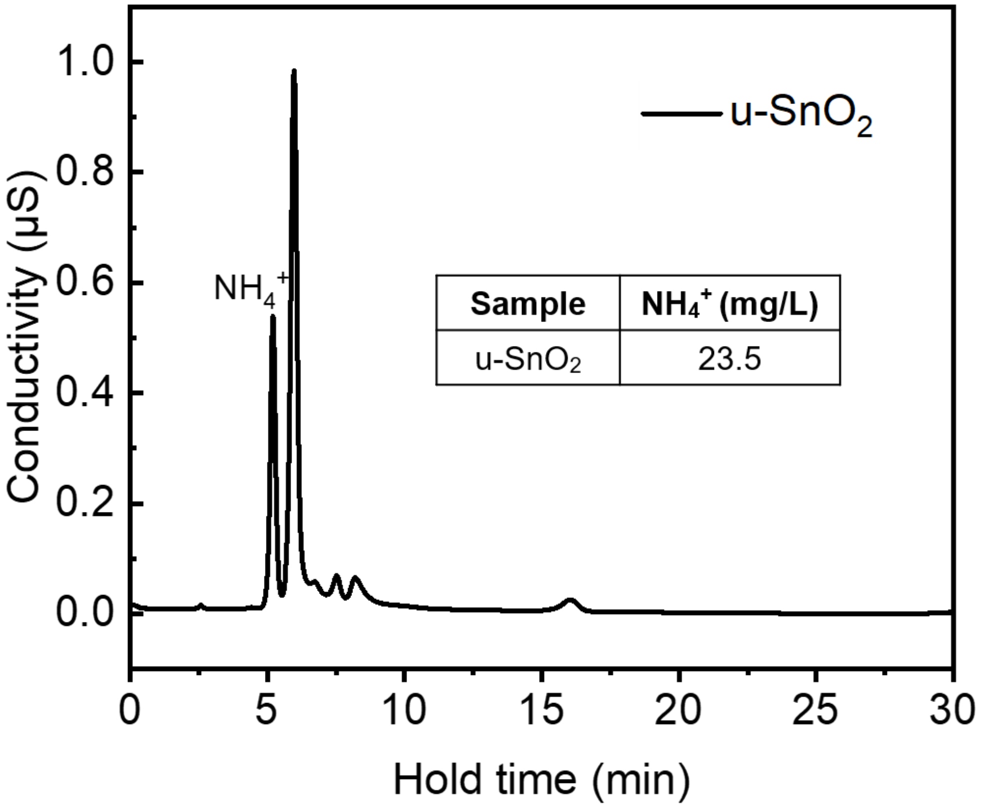

Figure S4. Ion chromatogram for the precursor solution after reaction a few hours.

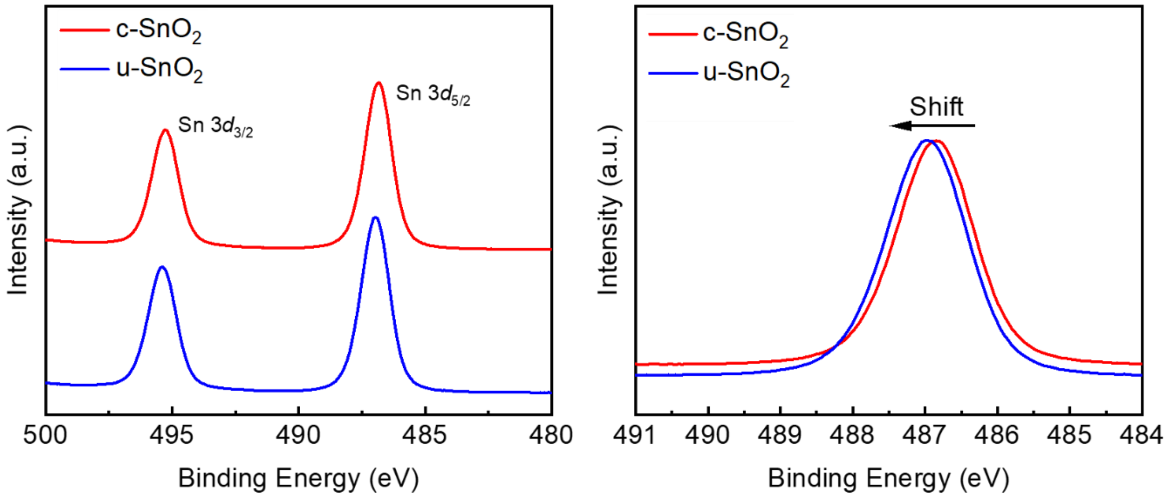

Figure S5. High-resolution XPS spectra of Sn 3$d_{3/2}$ and 3$d_{5/2}$ orbitals for c-SnO_2 and u-SnO_2.

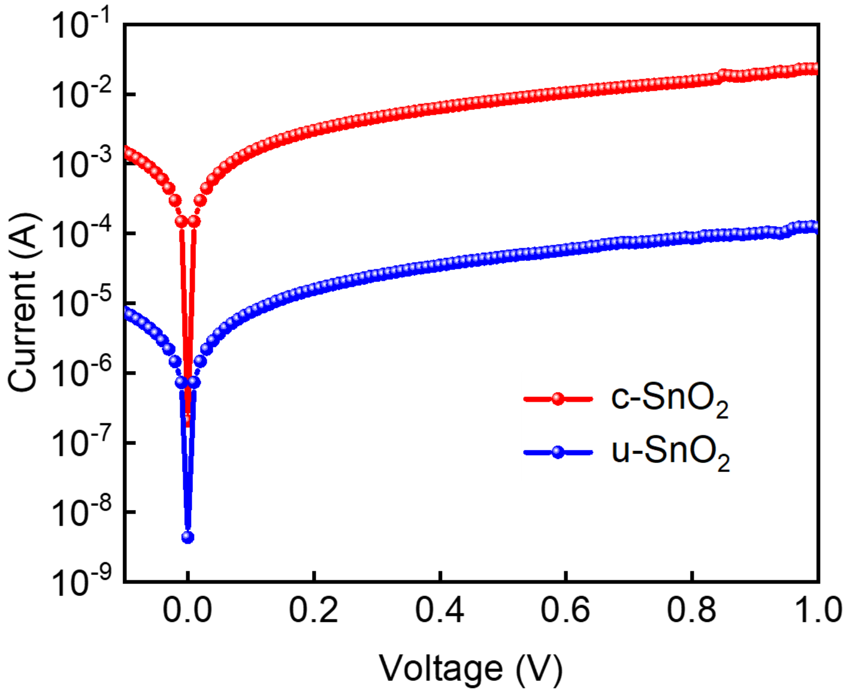

Figure S6. Dark current curves of the PSCs with different SnO_2 films.

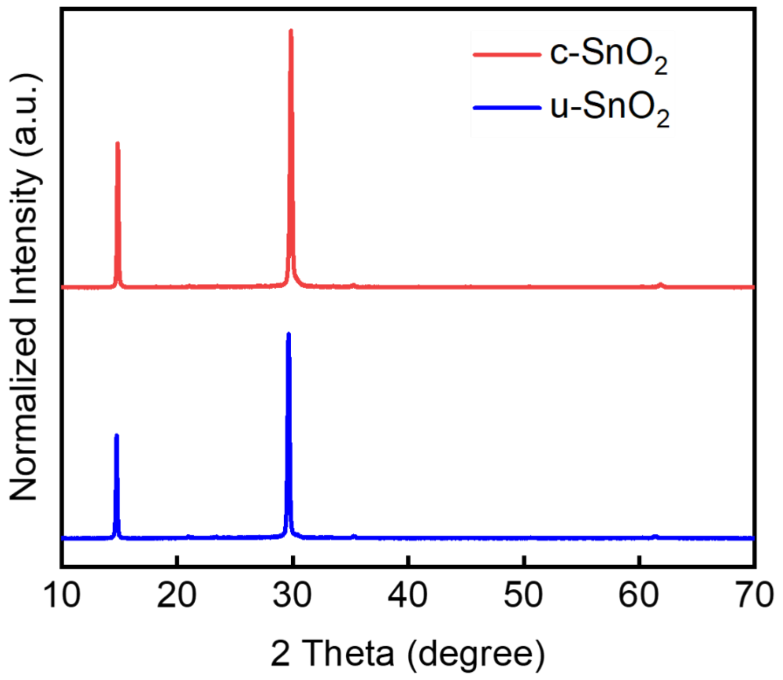

Figure S7. XRD patterns for $CsPbI_2Br$ films coated on different SnO_2 films.

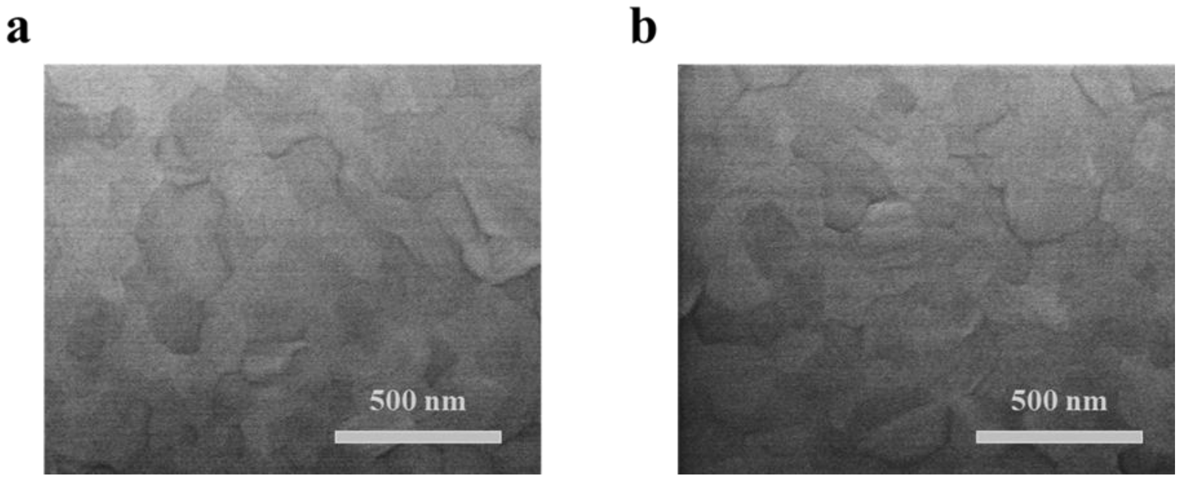

Figure S8. Top-view SEM images of $CsPbI_2Br$ films coated on (a) c-SnO_2 and (b) u-SnO_2 films.

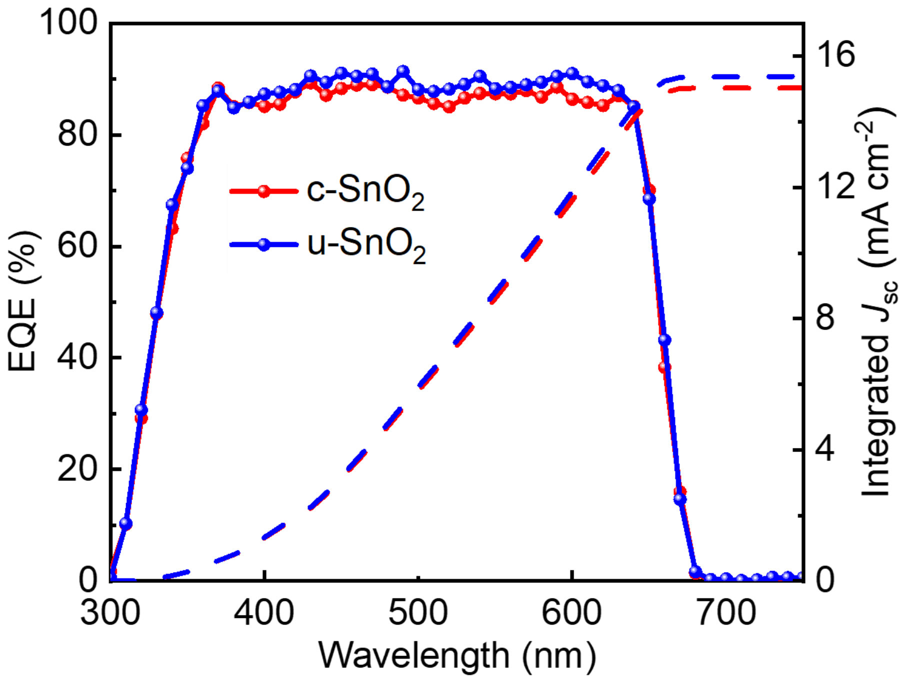

Figure S9. EQE spectra and integrated J_{SC} of as-fabricated solar cells.

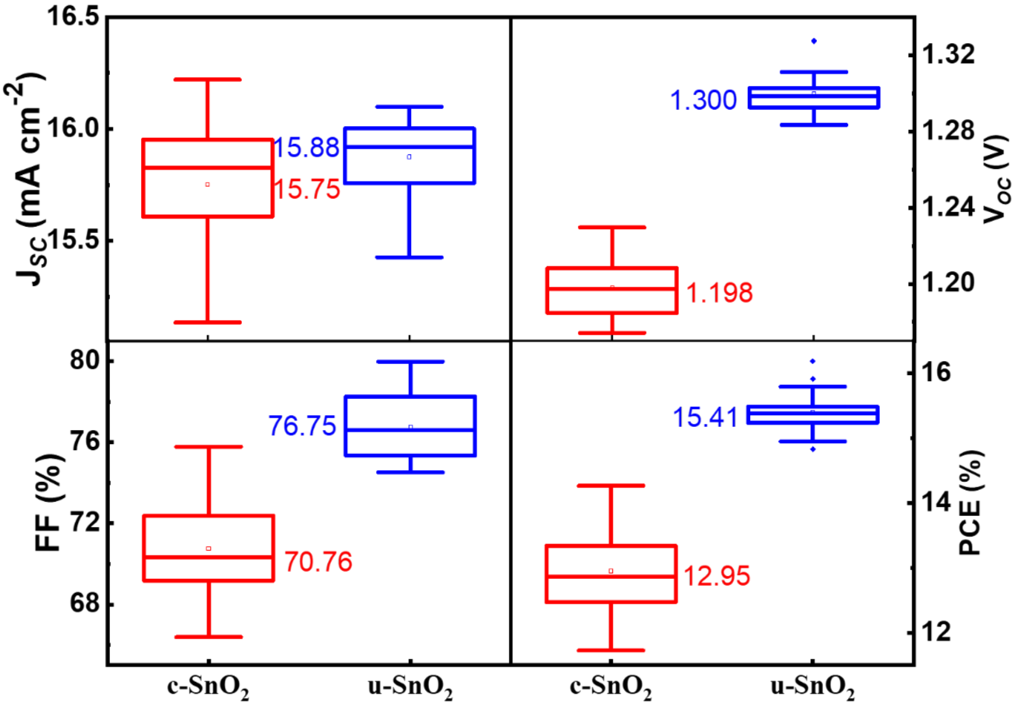

Figure S10. Statistical comparison of J–V parameters of PSCs based on different SnO_2 films for 20 samples.

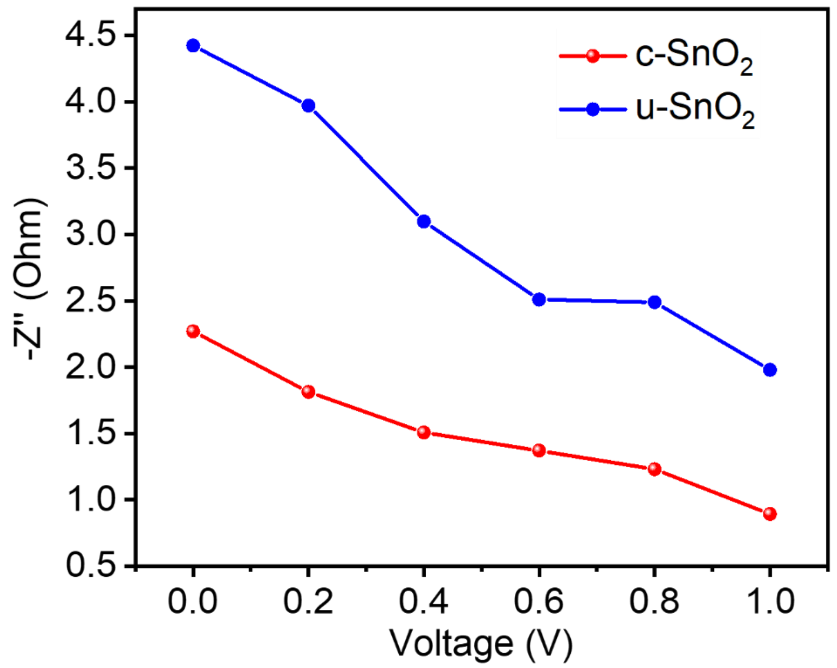

Figure S11. Recombination resistance as a function of applied bias voltage of solar cells based on different SnO_2 Films.

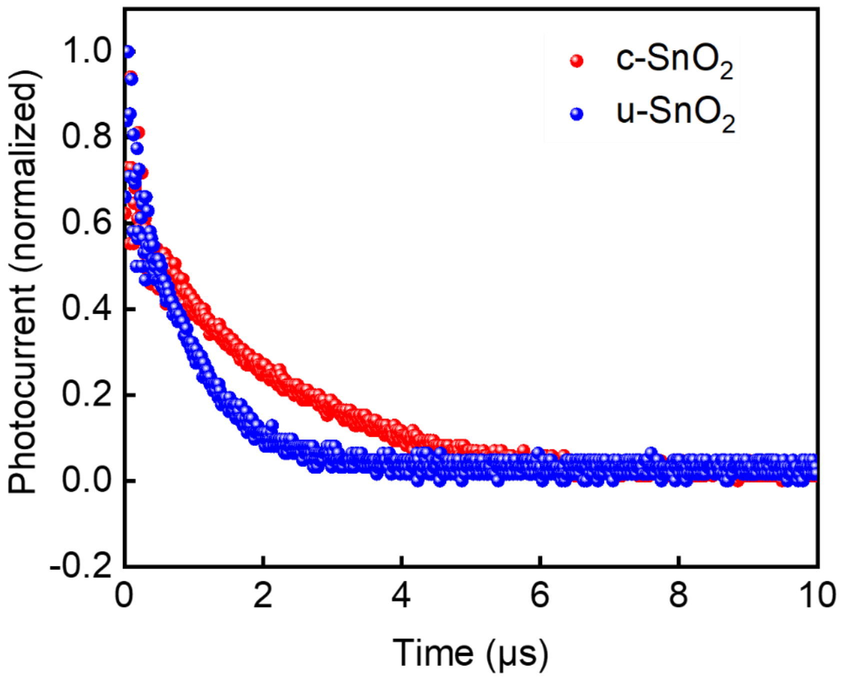

Figure S12. Transient photocurrent (TPC) decay curves of the PCS devices with different SnO_2 films.

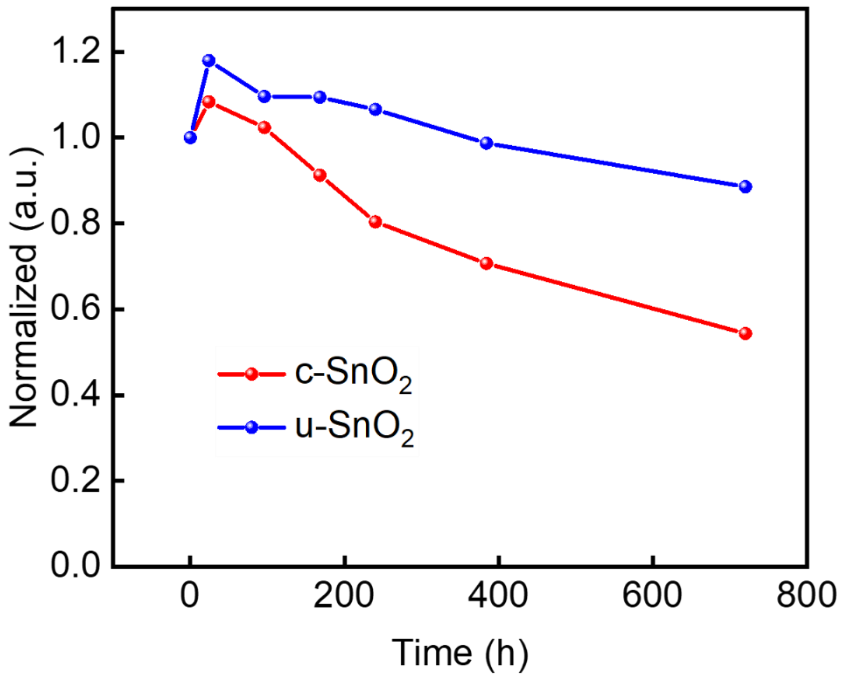

Figure S13. Thermal stability of PSCs with different SnO_2 films heated to 85 °C in N_2-filled glovebox.

Table S1. Summary of physical properties of c-SnO_2 and u-SnO_2 films.

**Characteristic**	I-V	Mott-Schottky		SCLC (interdigital electrodes)	
**Device**	ITO/SnO_2/Au	ITO/ SnO_2		ITO/SnO_2/Au	
**Structure**	σ (mS/cm)	φ_{bi} (V)	N_d (cm^{-3})	V_{TFL} (V)	N (cm^{-3})
**c-SnO_2**	0.95×10^{-2}	-0.75	2.33×10^{21}	26.52	1.68×10^{10}
**u-SnO_2**	1.48×10^{-2}	-0.95	2.72×10^{21}	5.75	3.68×10^{9}

Table S2. Photovoltaic parameters of pristine PSC based on different SnO_2 ETLs, measured under simulated AM 1.5G solar irradiation.

ETL	V_{OC} (mV)	J_{SC} (mA cm^{-2})	FF (%)	PCE (%)
**c-SnO_2**	1211.06	15.375	75.25	14.00
**u-SnO_2**	1300.07	15.676	79.56	16.22

Table S3. Performance parameters of $CsPbI_2Br$ perovskite solar cells with SnO_2 ETLs.

Device Structure	V_{OC} (V)	J_{SC} (mA cm^{-2})	FF (%)	PCE (%)	Ref.
**ITO/SnO_2/ZnO/Pero/Spiro-OMeTAD/MoO_3/Ag**	1.23	15.0	78.8	14.6	[1]
**ITO/SnO_2/Pero/CsBr/Spiro-OMeTAD/Au**	1.27	16.72	77.2	16.37	[2]
**ITO/SnO_2/PN4N/Pero/PDCBT/MoO_3/Ag**	1.30	15.3	81.5	16.2	[3]
**ITO/SnO_2/Aged-SnO_x/Pero/Poly(DTSTPD-r-BThTPD)/Au**	1.41	14.25	77	15.53	[4]
**ITO/SnO_2/SnO_x/Pero/PDTDT/Au**	1.42	15.02	81.29	17.36	[5]
**ITO/SnO_2 NPs/Pero/P3HT/Ag**	1.30	15.68	79.56	16.22	**This work**

4. References

[1] L. Yan, Q. Xue, M. Liu, Z. Zhu, J. Tian, Z. Li, Z. Chen, Z. Chen, H. Yan, H.-L. Yip, Y. Cao, *Adv. Mater.* **2018**, *30*, 1802509.

[2] Y. Zhang, C. Wu, D. Wang, Z. Zhang, X. Qi, N. Zhu, G. Liu, X. Li, H. Hu, Z. Chen, L. Xiao, B. Qu, *Solar RRL* **2019**, *3*, 1900254.

[3] J. Tian, Q. Xue, X. Tang, Y. Chen, N. Li, Z. Hu, T. Shi, X. Wang, F. Huang, C. J. Brabec, H.-L. Yip, Y. Cao, *Adv. Mater.* **2019**, *31*, 1901152.

[4] Z. Guo, A. K. Jena, I. Takei, G. M. Kim, M. A. Kamarudin, Y. Sanehira, A. Ishii, Y. Numata, S. Hayase, T. Miyasaka, *J. Am. Chem. Soc.* **2020**, *142*, 9725-9734.

[5] Z. Guo, A. K. Jena, I. Takei, M. Ikegami, A. Ishii, Y. Numata, N. Shibayama, T. Miyasaka, *Adv. Funct. Mater.* **2021**, *31*, 2103614
